# The Development of a Comprehensive Clinicopathologic Registry for Glomerular Diseases Using Natural Language Processing

**DOI:** 10.1177/20543581231178963

**Published:** 2023-06-16

**Authors:** Bryce Barr, Oksana Harasemiw, Ian W Gibson, Olivier Tremblay-Savard, Navdeep Tangri

**Affiliations:** 1 Department of Internal Medicine, University of Manitoba, Winnipeg, Canada; 2 Chronic Disease Innovation Centre, Seven Oaks General Hospital, Winnipeg, MB, Canada; 3 Department of Pathology, University of Manitoba, Winnipeg, Canada; 4 Shared Health Services Manitoba, Winnipeg, Canada; 5 Department of Computer Science, University of Manitoba, Winnipeg, Canada

**Keywords:** glomerulonephritis, Manitoba, clinical epidemiology

## Abstract

**Background:**

Glomerulonephritis (GN) represents a common cause of chronic kidney disease, and treatment to slow or prevent progression of GN is associated with significant morbidity. Large patient registries have improved the understanding of risk stratification, treatment selection, and definitions of treatment response in GN, but can be resource-intensive, with incomplete patient capture.

**Objective:**

To describe the creation of a comprehensive clinicopathologic registry for all patients undergoing kidney biopsy in Manitoba, using natural language processing software for data extraction from pathology reports, as well as to describe cohort characteristics and outcomes.

**Design:**

Retrospective population-based cohort study.

**Setting:**

Tertiary care center in the province of Manitoba.

**Patients:**

All patients undergoing a kidney biopsy in the province of Manitoba from 2002 to 2019.

**Measurements:**

Descriptive statistics are presented for the most common glomerular diseases, along with outcomes of kidney failure and mortality for the individual diseases.

**Methods:**

Data from native kidney biopsy reports from January 2002 to December 2019 were extracted into a structured database using a natural language processing algorithm employing regular expressions. The pathology database was then linked with population-level clinical, laboratory, and medication data, creating a comprehensive clinicopathologic registry. Kaplan-Meier curves and Cox models were constructed to assess the relationship between type of GN and outcomes of kidney failure and mortality.

**Results:**

Of 2421 available biopsies, 2103 individuals were linked to administrative data, of which 1292 had a common glomerular disease. The incidence of yearly biopsies increased almost 3-fold over the study period. Among common glomerular diseases, immunoglobulin A (IgA) nephropathy was the most common (28.6%), whereas infection-related GN had the highest proportions of kidney failure (70.3%) and all-cause mortality (42.3%). Predictors of kidney failure included urine albumin-to-creatinine ratio at the time of biopsy (adjusted hazard ratio [HR] = 1.43, 95% confidence interval [CI] = 1.24-1.65), whereas predictors of mortality included age at the time of biopsy (adjusted HR = 1.05, 95% CI = 1.04-1.06) and infection-related GN (adjusted HR = 1.85, 95% CI = 1.14-2.99, compared with the reference category of IgA nephropathy).

**Limitations:**

Retrospective, single-center study with a relatively small number of biopsies.

**Conclusions:**

Creation of a comprehensive glomerular diseases registry is feasible and can be facilitated through the use of novel data extraction methods. This registry will facilitate further epidemiological research in GN.

## Abrégé

**Contexte:**

La glomérulonéphrite (GN) est une cause courante d’insuffisance rénale chronique. Le traitement pour ralentir ou prévenir la progression de la GN est associé à une morbidité significative. Les vastes registres de patients ont permis de mieux comprendre la stratification des risques, la sélection du traitement et les définitions de la réponse au traitement en contexte de GN, mais ces registres exigent beaucoup de ressources et la saisie des données du patient peut être incomplète.

**Objectifs:**

Décrire, à l’aide d’un logiciel de traitement en langage naturel pour l’extraction des données des rapports pathologiques, la création d’un registre clinicopathologique complet pour tous les patients subissant une biopsie rénale au Manitoba, et décrire les caractéristiques et les résultats de la cohorte.

**Conception:**

Étude de cohorte rétrospective basée sur la population.

**Cadre:**

Centre de soins tertiaires de la province du Manitoba.

**Sujets:**

Tous les patients ayant subi une biopsie rénale entre 2002 et 2019 au Manitoba.

**Mesures:**

Des statistiques descriptives sont présentées pour les maladies glomérulaires les plus courantes, accompagnées des résultats d’insuffisance rénale et de mortalité pour les maladies individuelles.

**Méthodologie:**

Les données des rapports de biopsie sur un rein natif entre janvier 2002 et décembre 2019 ont été extraites d’une base de données structurée à l’aide d’un algorithme de traitement en langage naturel utilisant des expressions régulières. La base de données des pathologies a ensuite été reliée aux données cliniques, de laboratoire et de médicaments au niveau de la population, pour créer un registre clinicopathologique complet. Des courbes Kaplan-Meier et des modèles de Cox ont été construits pour évaluer la relation entre le type de GN et les résultats d’insuffisance rénale et de mortalité.

**Résultats:**

Des 2 421 biopsies réalisées, 2 103 concernaient des individus liés à des données administratives; de ceux-ci, 1 292 étaient atteints d’une maladie glomérulaire courante. L’incidence des biopsies annuelles a presque été multipliée par trois au cours de la période étudiée. Parmi les maladies glomérulaires courantes, la néphropathie à IgA était la plus fréquente (28,6 %). Les GN liées à une infection présentaient les proportions les plus élevées d’insuffisance rénale (70,3 %) et de mortalité toutes causes confondues (42,3 %). Les facteurs prédictifs de l’insuffisance rénale comprenaient le rapport albumine/créatinine urinaire au moment de la biopsie (rapport de risque corrigé [RRc]: 1,43; intervalle de confiance à 95 % [IC 95 %]: 1,24-1,65), alors que les facteurs prédictifs de la mortalité incluaient l’âge au moment de la biopsie (RRc: 1,05; IC 95 %: 1,04-1,06) et les GN liées à une infection (RRc: 1,85; IC 95 %: 1,14-2,99; par rapport à la catégorie de référence de la néphropathie à IgA).

**Limites:**

Étude rétrospective menée dans un seul centre sur un nombre relativement faible de biopsies.

**Conclusion:**

La création d’un registre complet des maladies glomérulaires est réalisable et peut être facilitée par l’utilisation de nouvelles méthodes d’extraction des données. Ce registre facilitera de futures recherches épidémiologiques sur la GN.

**Enregistrement de l’essai:**

ne s’applique pas, cette étude n’est pas un essai clinique.

## Introduction

Glomerulonephritis (GN) are a heterogeneous group of rare immune-mediated kidney diseases that are associated with significant morbidity and mortality.^1-3^ The treatments aimed at preventing kidney failure in this population are also a cause of significant morbidity,^4-6^ and thus it is important to identify risk factors for progression to develop effective therapies while limiting toxicity. Unfortunately, the rare nature of these diseases has impaired advancements in the epidemiological understanding and pharmacological treatment of GN, which in turn has limited our ability to slow disease progression with more tolerable treatments.^7^ It is important to note that although these diseases are individually uncommon,^8^ they collectively affect millions of individuals worldwide and are the most common cause of kidney failure requiring kidney replacement therapy and/or transplantation in individuals below the age of 50.^9-11^ Therefore, in addition to being an important cause of morbidity and mortality, GN has a significant adverse economic and personal impact in society.

The establishment of large patient registries, designed to capture all patients with GN in a given geographic region, have been successful in improving our understanding of the natural history of GN and with identifying potential participants for clinical trials. A national survey of Canadian nephrologists indicated that most nephrologists support the establishment of GN registries and that they would be willing to enroll their patients in such a registry.^12^ Furthermore, registries provide an opportunity for collaboration between clinicians and researchers as well as different centers.^13-15^ Fortunately, a kidney biopsy is typically required for the accurate diagnosis of GN, and therefore it is possible to identify most individuals in a particular location who have biopsy-proven GN. In Manitoba, all kidney pathology data since 2002 are readily available in the form of individual kidney biopsy reports, with findings on light microscopy, immunofluorescence microscopy, and electron microscopy reported for each biopsy and summarized in a single report. In addition, these data can be linked to administrative, laboratory, medication, and hospital data via the Manitoba Centre for Health Policy (MCHP), to form a comprehensive clinicopathologic registry. In this article, we discuss the creation and aims of the Manitoba Glomerular Diseases Registry, and the baseline demographic and outcome (kidney failure and mortality) data for biopsy-diagnosed kidney disease in the province of Manitoba.

## Methods

### Creation of the Registry

Since 2002, all kidney biopsies performed in Manitoba have been interpreted and reported at a single site—the Health Sciences Centre in Winnipeg, Manitoba, Canada. The biopsy reports are stored at the Department of Pathology as individual Microsoft Word and PDF documents, and each report contains demographic data (date of biopsy, date of birth, sex, and personal health identification numbers [PHINs]) as well as comprehensive pathological data. We extracted data from all biopsy reports from all patients who underwent a native kidney biopsy in the province for any clinical indication between January 1, 2002, and December 31, 2019, excluding patients who underwent transplant or donor implantation kidney biopsies.

A list of data extracted is presented in Supplementary Table S1. Data were extracted and outputted in a structured tabular format that could easily be reviewed by members of the research team and that could be parsed computationally for linkages with other databases, and for further downstream analyses. For this reason, the CSV (comma-separated values) format was selected for the output, as it is a very simple tabular format that can be recognized by Microsoft Excel. Below is a description of the approach used to extract data.

### Data Extraction Methodology—Biopsy Reports

Whereas PDF documents only contained textual information, the Microsoft Word documents often contained pictures, in which patient information (name, age, etc.) was presented within. The information stored in the biopsy reports was semi-structured, in the sense that most reports contained the same main sections, namely, patient information, gross description, microscopic description, immunofluorescence, electron microscopy, and diagnosis. However, these sections were not always presented in the same order, some were absent, and the data within the sections were presented in highly variable language.

### Data Extraction Process—Text Extraction

To automate the extraction of the data from the biopsy reports, the Python language was selected to build the application. The tika-python library was used.^16^ This is a Python port of the Apache Tika library^17^ that is built to extract text from different types of files (eg, PDF and Word files). The Tesseract optical character recognition engine^18^ was installed and used in conjunction with Tika to extract the text from the figures in the Word documents.

### Regular Expressions

The main tool used to extract all the relevant information from the biopsy reports is called *regular expressions*. Regular expressions, also called *regexes* for short, are rooted in theoretical mathematics and computer science.^19,20^ Regular expressions nowadays are available in many popular programming languages (including Python, the language selected for this project). A regular expression is made up of metacharacters (these act as the rules and instructions of the regular expression to ensure the desired data are retrieved) and literal characters (the actual letters or words being looked for). It helps to see regexes as a language^21^: In this context, literal characters can be seen as words and metacharacters as grammar. A regular expression can be designed to search, in a generalized way, for all data that match a specific pattern in the text.

The data that needed to be extracted could take many forms and have a range of possible values. A specific regular expression was designed to recognize and extract each of these data points, considering both the type of data (numeric, text, binary, etc) and the text that is usually in its vicinity. The latter was not necessarily trivial, as the same information could be presented in many ways (eg, “There are 10 globally sclerosed glomeruli” vs “The number of globally sclerotic glomeruli is 10” vs “Ten glomeruli are globally sclerosed”). The challenge was to strike a balance between designing regular expressions that were specific enough to collect only the information of interest (without confusion) and general enough to recognize all textual variations that appeared in the different biopsy reports.

### Development Process

An iterative development strategy was used to train the algorithm. This began with 10 annotated biopsies indicating the desired output for each variable from which the algorithm was developed. The algorithm was refined using another sample of 20 biopsies, followed by a third test of 50 biopsies, with biopsies specifically selected to represent a range of pathological diagnoses and microscopic features as well as varying times within the study period. Errors in the algorithm output were identified in each iteration and subsequently corrected. Following the third iteration, the algorithm was 100% accurate in identifying all variables apart from primary and secondary diagnosis. Therefore, the algorithm was applied to all available biopsy reports, creating the pathology registry. A random audit was conducted on a total of 100 biopsies by one of the authors (a GN specialist), with each biopsy analyzed in its entirety for the fidelity of the algorithm output. At least 5 biopsies from each year in the study period were included in the audit. The output of light microscopic, immunofluorescence, and electron microscopic features was without error. In one biopsy with a diagnosis of lupus nephritis (LN), the algorithm omitted the activity and chronicity indices; these were, therefore, independently verified for each biopsy demonstrating LN. Similarly, in one biopsy with a diagnosis of IgA nephropathy, the algorithm incorrectly reported the Oxford score; Oxford scores were, therefore, independently verified in the individual biopsy reports where applicable. In addition to the random audit, each primary and secondary diagnosis was independently verified on all 2421 biopsies.

### Linkage With Administrative Data

Once the biopsy data were extracted into a single CSV file, a data linkage was created with the Population Health Research Data Repository at the MCHP^22^ using each individual’s personal provincial health identification number to create a comprehensive clinicopathologic registry. Manitoba Health provides health care services to Manitobans through a single-payer public health care system, resulting in near-complete claims data available for the majority of the population. Databases linked were the Manitoba Health Insurance Registry (patient registry and coverage dates), Medical Services (physician claims data), Canadian Institute for Health Information (CIHI) Discharge Abstract Database (DAD) (hospital admissions data), the Shared Health Diagnostic Services database (laboratory diagnostics), the Drug Program Information Network (DPIN) database (drug prescriptions), Vital Statistics (date and causes(s) of death), and Canada Census data (to ascertain socioeconomic status and dwelling location). Descriptions of databases and years accessed are presented in Supplementary Table S2. The creation of the registry and the linkage was approved by the University of Manitoba Health Research Ethics Board (HS24601(H2021:031)), and by each of the data providers.

### Study Population—Description of Baseline Characteristics and Outcomes

In this article, we present the incidence and prevalence of glomerular disease, along with other nonglomerular kidney diseases based on kidney biopsy diagnosis in Manitoba, using a retrospective, population-based cohort design. Demographic, clinical data, including baseline laboratory results, comorbidities, and prescriptions of disease-modifying medications, are presented.

### Categories of Disease

Diseases were initially classified into 7 broad groups, in an approach described previously.^23,24^ The groups include proliferative glomerulonephritis, nonproliferative glomerulonephritis, diabetic nephropathy, tubulointerstitial disease, vascular disease, deposition diseases, and hereditary kidney diseases. Patients with a normal or nondiagnostic biopsy due to inadequate sampling of kidney tissue were not classified. A full list of proliferative and nonproliferative glomerulonephritis included can be found in Supplementary Table S5. Diabetic nephropathy was considered on its own, separate from other nonproliferative glomerulonephritis. Tubulointerstitial diseases included acute tubular injury, acute and chronic interstitial nephritis, light chain cast nephropathy, and light chain proximal tubulopathy. Vascular diseases included thrombotic microangiopathy and ischemic glomerulopathy/hypertensive kidney disease. Deposition diseases include AL amyloidosis and monoclonal immunoglobulin deposition disease. Hereditary diseases included Alport syndrome/hereditary nephritis, Fabry disease, and thin basement membrane nephropathy. In Table 1, we present data for the most common individual glomerular diseases found in the registry (focal segmental glomerulonephritis [FSGS], secondary FSGS, IgA nephropathy, infection-related GN [IRGN], LN, membranous nephropathy, pauci-immune GN, and minimal change disease).

**Table 1.** Demographic and Clinical Characteristics at the Time of Biopsy for Individuals Who Underwent a Kidney Biopsy in Manitoba Between January 1, 2002, and December 31, 2019, and Who Have Select Types of GN (N = 1299 Biopsies; 1292 Individuals).

	Primary diagnostic category^a^							
	FSGS	Secondary FSGS	IgA nephropathy	Infection-related GN	Lupus nephritis	Membranous nephropathy	Pauci-immune GN	Minimal change disease
N (%)	115 (8.9)	127 (9.8)	371 (28.6)	111 (8.6)	132 (10.2)	179 (13.8)	203 (15.6)	61 (4.7)
Year of diagnosis								
2002-2006	22 (19.3)	20 (15.8)	67 (18.1)	13 (11.7)	20 (15.2)	37 (20.7)	41 (20.2)	13 (21.3)
2007-2012	46 (40.0)	43 (33.9)	99 (26.7)	27 (24.3)	50 (37.9)	57 (31.8)	64 (31.5)	11 (18.0)
2013-2019	47 (40.9)	64 (50.4)	205 (55.3)	71 (64.0)	62 (47.0)	85 (47.5)	98 (48.3)	37 (60.7)
Age, years	51.2 ± 17.3	51.1 ± 16.0	43.1 ± 15.5	50.5 ± 15.5	38.3 ± 15.4	56.1 ± 14.0	60.7 ± 15.8	52.0 ± 17.6
Female sex	46 (40.0)	39 (30.7)	146 (39.4)	37 (33.3)	113 (85.6)	66 (36.9)	103 (50.7)	30 (49.2)
eGFR, mL/ min/1.73 m^2^	50.0 (35.0-86.0)	35.5 (23.0-59.0)	45.0 (18.0-78.0)	15.0 (9.0-41.0)	65.0 (33.0-111.0)	80.0 (49.0-99.0)	19.0 (10.0-29.0)	58.0 (34.0-103.0)
Urine ACR, mg/mmol	386.9 (179.2, 619.5)	169.5 (96.3-253.9)	166.8 (90.6-328.2)	272.0 (130.5-459.0)	210.0 (65.9-426.4)	385.4 (219.1-637.7)	129.1 (57.4-278.3)	509.8 (198.9-657.6)
Dwelling location								
Rural	43 (37.7)	38 (29.9)	143 (38.5)	66 (59.5)	55 (42.0)	64 (35.8)	106 (52.5)	18 (29.5)
Urban	71 (62.3)	89 (70.1)	228 (61.5)	45 (40.5)	76 (58.0)	115 (64.3)	96 (47.5)	43 (70.5)
Socioeconomic status								
1 (Lowest)	29 (25.4)	31 (24.4)	118 (31.8)	56 (50.5)	35 (26.7)	44 (24.6)	55 (27.2)	15 (24.6)
2	21 (18.4)	26 (20.5)	75 (20.2)	25 (22.5)	27 (20.6)	38 (21.2)	41 (20.3)	12 (19.7)
3	15 (13.2)	33 (26.0)	66 (17.8)	10 (9.0)	21 (16.0)	22 (12.3)	31 (15.4)	9 (14.8)
4	27 (23.7)	21 (16.5)	76 (20.5)	12 (10.8)	29 (22.1)	40 (22.4)	40 (19.8)	8 (13.1)
5 (Highest)	22 (19.3)	16 (12.6)	36 (9.7)	8 (7.2)	19 (14.5)	35 (19.6)	35 (17.3)	17 (27.9)
Medications								
Anti-blood pressure medications *(not including ACEi/ARBs)*	93 (80.9)	91 (71.7)	218 (58.8)	91 (82.0)	71 (53.8)	135 (75.4)	135 (66.5)	46 (75.4)
ACEi/ARBs	88 (76.5)	91 (71.7)	255 (68.7)	30 (27.0)	68 (51.5)	151 (84.4)	44 (21.7)	30 (49.2)
Statins	50 (43.5)	50 (39.4)	84 (22.6)	34 (30.6)	18 (13.6)	108 (60.3)	38 (18.7)	14 (23.0)
Comorbidities^b^	1.0 (0.0-2.0)	1.0 (0.0-2.0)	1.0 (0.0-2.0)	3.0 (1.0-5.0)	1.0 (1.0-2.0)	1.0 (0-2.0)	2.0 (1.0-3.0)	0 (0, 1.0)
Follow-up time (years)^c^	7.3 (3.5-11.8)	6.1 (3.8-11.0)	5.7 (3.1-10.9)	3.9 (2.4-6.9)	6.6 (3.5-10.1)	5.9 (2.9-10.3)	4.9 (1.6-8.6)	6.7 (2.9-11.5)
All-cause mortality	26 (22.6)	29 (22.8)	67 (18.1)	47 (42.3)	24 (18.2)	36 (20.1)	81 (39.9)	7 (11.5)
Kidney failure^d^	32 (27.8)	55 (43.3)	167 (45.0)	78 (70.3)	44 (33.3)	24 (13.4)	107 (52.7)	*suppressed*

*Note.* Data presented as N (%) for categorical variables, mean ± standard deviation for normally distributed continuous variables, and median (interquartile range) for non-normally distributed continuous variables, as appropriate. Urine ACR values include urine protein-to-creatinine ratio tests converted to urine ACR using the equation developed by Weaver et al^25^. Cell sizes <6 are suppressed to protect individual anonymity. Labs values were assessed ±3 months of biopsy, and medications were assessed within 6 months post-biopsy. GN = glomerulonephritis; FSGS = focal segmental glomerulosclerosis; eGFR = estimated glomerular filtration rate; ACR = albumin-to-creatinine ratio; ACEi = angiotensin-converting enzyme inhibitor; ARB = angiotensin receptor blocker.

a Indivdiuals may be included in more than one category if they had multiple biopsies where the subsequent biopsy revealed a different primary diagnosis.

b Comorbid conditions were based on the weighted Charlson Comorbidity Index and were drawn from data collected through medical claims and hospital admission data during the 3 years prior to the date of biopsy.

c Individuals were followed until they reached the end of the study period (March 31, 2021), lost provincial health coverage, migrated from the province, died, or had a second follow-up biopsy that resulted in a different primary diagnosis.

d Kidney failure defined as requiring dialysis or kidney transplantation.

For individuals with multiple biopsies, if the initial biopsy was nondiagnostic, the index date was the first diagnostic biopsy. Conversely, if the initial biopsy was diagnostic, but a subsequent biopsy was nondiagnostic or showing sclerosis from the prior diagnosis, those individuals were classified by their initial diagnostic biopsy. For individuals with the same diagnosis on multiple biopsies, only the index biopsy was considered in the analysis. In some cases, the initial biopsy diagnosis (eg, membranous nephropathy) was followed up by a subsequent biopsy showing a very different diagnosis (eg, pauci-immune glomerulonephritis). In this instance, such individuals were counted in multiple categories and censored at the time of follow-up biopsy. For individuals with a biopsy identifying multiple disease processes (eg, infection-related glomerulonephritis on a background of diabetic nephropathy), the acute process was considered the primary diagnosis, and the background pathology the secondary diagnosis.

### Covariates

Comorbid conditions were assessed using the weighted Charlson Comorbidity Index and were drawn from data collected through medical claims and hospital admission data during the 3 years prior to date of biopsy. Socioeconomic status (quintiles 1-5, from lowest to highest respectively) and dwelling location (urban vs rural) were derived from dissemination area–level information generated from the Canadian Census. Laboratory values were assessed ±3 months from the date of biopsy, and medications were assessed within 6 months post-biopsy. Medications were classified according to the Anatomical Therapeutic Chemical system (Supplementary Table S4).

### Outcomes

The main outcomes of interest include all-cause mortality and kidney failure and were compared between glomerular diseases. Kidney failure was defined as dialysis or kidney transplant; administrative data definitions are presented in Supplementary Table S3. All-cause mortality was ascertained using the Manitoba Health Registry. Individuals were followed until they reached an outcome, migrated out of the province, lost provincial health coverage, underwent a second biopsy that resulted in a different diagnosis, or reached the study end date (March 31, 2021).

### Statistical Analysis

Descriptive statistics, presented for the most frequently diagnosed glomerular diseases in the cohort, hereafter referred to as “common GN,” are expressed as mean ± standard deviation for normal continuous variables, median and interquartile ranges for non-normal continuous variables, and percentages for categorical variables. We constructed Kaplan-Meier curves for all-cause mortality and kidney failure. Cox proportional hazard models were developed to analyze the association between disease type and the outcomes of all-cause mortality and kidney failure. Individuals who had multiple biopsies that resulted in different diagnoses were excluded from the Cox models and Kaplan-Meier curves. Only complete cases were included in the analyses.

### Subanalyses

For all 7 broad categories of kidney diseases, including nonglomerular diseases, we present descriptive statistics, expressed as mean ± standard deviation for normal continuous variables, median and interquartile ranges for non-normal continuous variables, and percentages for categorical variables.

## Results

### Baseline Characteristics

**Figure 1.**
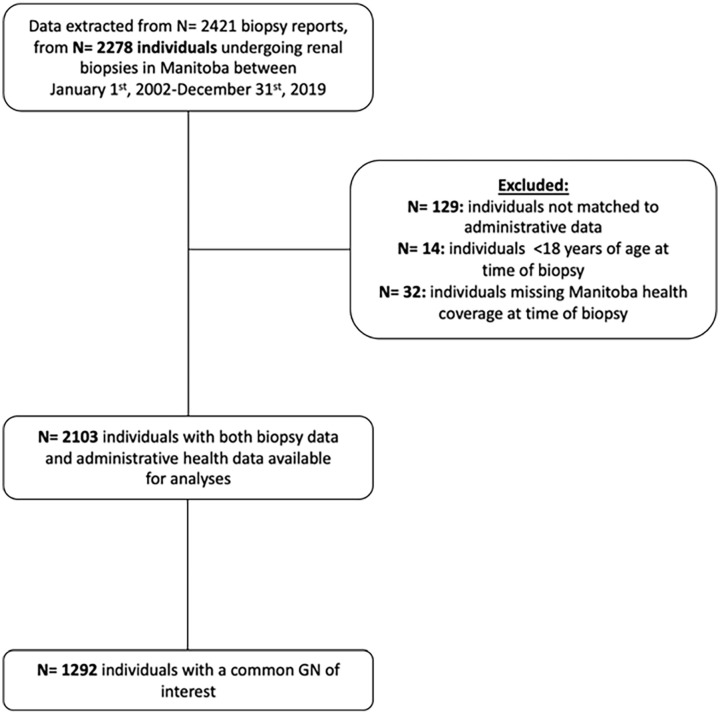
Cohort flow diagram.

**Figure 2.**
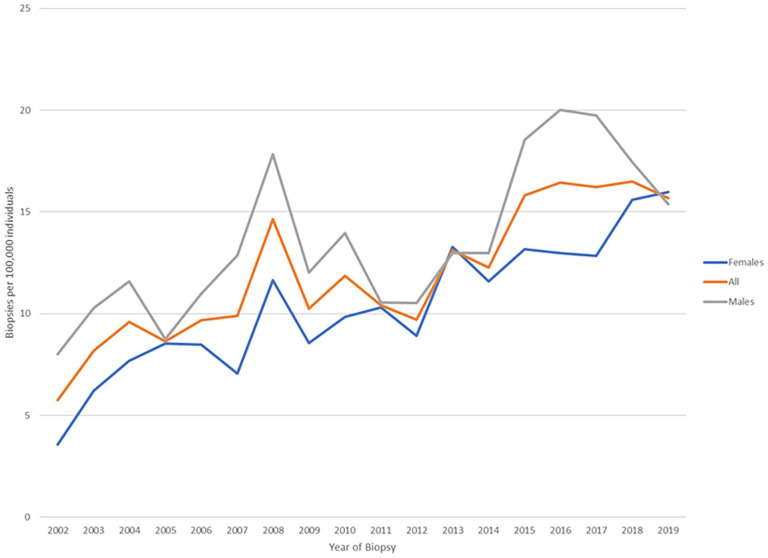
Incidence of kidney biopsies conducted in Manitoba from 2002 to 2019.

**Figure 3.**
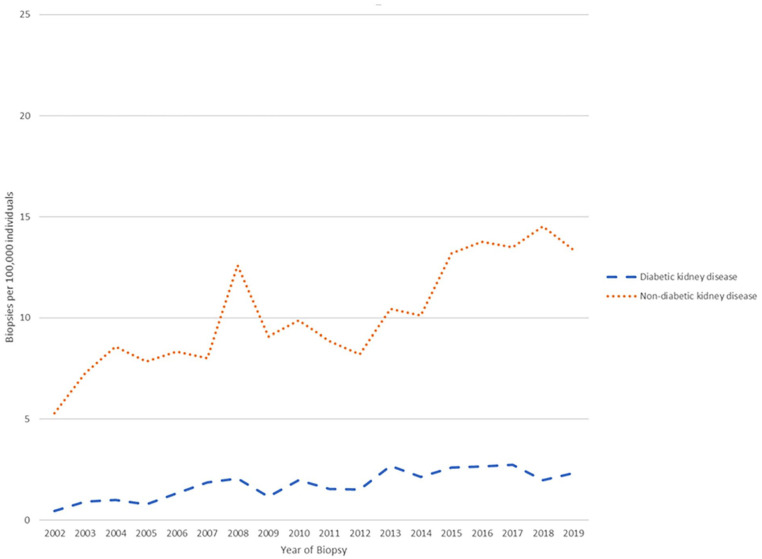
Incidence of biopsy-proven diabetic and nondiabetic kidney disease from 2002 to 2019.

A total of 2421 native kidney biopsy reports between January 1, 2002, and December 31, 2019, were available, from 2278 individual patients. Of these, 2103 individuals were linked to administrative data; of these individuals, 1292 had a common GN and were included in our main analyses (Figure 1). Of the total number of biopsies performed, 57 biopsies were either normal or nondiagnostic (2.7%). The incidence of biopsy increased from 5.7 per 100 000 individuals in 2002 to 15.7 per 100 000 individuals in 2019 (Figure 2). The incidence of biopsy demonstrating nondiabetic kidney disease increased from 5.2 per 100 000 individuals to 13.4 per 100 000 individuals over the course of the study period, whereas the incidence of biopsy demonstrating diabetic nephropathy increased from 0.5 per 100 000 individuals to 2.3 per 100 000 individuals (Figure 3).

The most frequent GN was IgA nephropathy (n = 371, 30%), with pauci-immune glomerulonephritis being second most common (n = 203, 16.4%). This was true across all time periods in the study. Patients with a diagnosis of LN tended to be younger, whereas patients with pauci-immune GN tended to be older. Patients with IRGN and pauci-immune GN had the lowest median estimated glomerular filtration rate (eGFR) at the time of biopsy, whereas patients with membranous nephropathy and focal segmental glomerulosclerosis (FSGS) had the highest median urine albumin-to-creatinine ratio (ACR). Patients with IRGN had the highest number of comorbidities. The baseline characteristics of the patients with common GNs are presented in Table 1.

### Kidney Failure

The Kaplan-Meier curves for kidney failure are shown in Supplemental Figure 1. The IRGN group presented with the highest rate of kidney failure, with 70.3% progressing to kidney failure over a median of 3.9 years of follow-up. In contrast, the membranous nephropathy group had the lowest risk of kidney failure, with 13.4% progression to kidney failure over a median of 5.9 years of follow-up.

Compared with the reference group of IgA nephropathy, FSGS (HR = 0.54, 95% CI = 0.31-0.94), IRGN (HR = 0.65, 95% CI = 0.43-0.99), membranous nephropathy (HR = 0.52, 95% CI = 0.29-0.95), and minimal change disease (HR = 0.20, 95% CI = 0.06-0.63) were associated with a lower risk kidney failure, following adjustment for age, sex, baseline eGFR, and urine ACR. Increasing urine ACR (HR = 1.43, 95% CI = 1.24-1.65) was associated with a higher risk of kidney failure. Predictors for kidney failure and mortality are summarized in Table 2.

**Table 2.** Hazard Ratios and 95% Confidence Intervals for the Association Between Primary Diagnosis and the Outcomes of Mortality and Kidney Failure.

	Mortality^a^	Mortality^b^	Kidney failure^c^	Kidney failure^d^
FSGS	1.43 (0.82-2.50)	0.89 (0.49-1.61)	**0.49 (0.29-0.83)**	**0.54 (0.31-0.94)**
Secondary FSGS	1.43 (0.84-2.44)	0.88 (0.50-1.52)	0.97 (0.65-1.45)	1.08 (0.72-1.63)
IgA nephropathy^f^	—	—	—	—
Infection-related glomerulonephritis	**3.68 (2.29-5.90)**	**1.85 (1.14-2.99)**	**1.53 (1.02-2.30)**	**0.65 (0.43-0.99)**
Lupus nephritis	0.91 (0.49-1.67)	1.41 (0.75-2.63)	**0.52 (0.32-0.86)**	0.69 (0.41-1.14)
Membranous nephropathy	1.15 (0.68-1.93)	0.68 (0.38-1.24)	**0.28 (0.16-0.49)**	**0.52 (0.29-0.95)**
Pauci-immune glomerulonephritis	**2.45 (1.59-3.78)**	1.11 (0.70-1.77)	**1.55 (1.11-2.17)**	1.13 (0.79-1.62)
Minimal change disease	0.44 (0.14-1.41)	**0.29 (0.09-0.96)**	**0.16 (0.05-0.50)**	**0.20 (0.06-0.63)**
Age at biopsy		**1.05 (1.04-1.06)**		**0.98 (0.98-0.99)**
Sex^e^		1.24 (0.91-1.69)		0.93 (0.72-1.20)
eGFR		**0.99 (0.98-0.99)**		**0.97 (0.96-0.97)**
Log urine ACR		**1.32 (1.13-1.54)**		**1.43 (1.24-1.65)**
AIC	2334.25	2203.16	3280.97	3045.75
AUC	0.63	0.78	0.66	0.82

*Note.* N = 898 for mortality outcome; N = 891 for kidney failure outcome. Excluding those with >1 primary diagnosis. FSGS = focal segmental glomerulonephritis; eGFR = estimated glomerular filtration rate; ACR = albumin-to-creatinine ratio; AIC= Akaike information criterion; AUC = area under the curve.

a Model includes primary diagnosis category.

b Model includes primary diagnosis category, age, sex, eGFR, urine ACR.

c Model includes all primary diagnosis categories.

d Model includes all primary diagnosis categories, age, sex, eGFR, urine ACR.

e Reference category is females.

f Reference category is IgA nephropathy.Boldfaced values are those reaching statistical significance.

### Mortality

The Kaplan-Meier curves for all-cause mortality are shown in Supplemental Figure 2. The IRGN had the highest risk of all-cause mortality, with 42.3% of patients dying over a median of 3.9 years of follow-up, whereas IgA nephropathy had the lowest risk of all-cause mortality (18.1%) over a median of 5.7 years of follow-up. After adjustment for age, sex, baseline eGFR, and urine ACR, a diagnosis of IRGN (HR = 1.85, 95% CI = 1.14-2.99) and increasing age (HR = 1.05, 95% CI = 1.04-1.06) were associated with a higher risk of mortality. Conversely, higher baseline eGFR (HR = 0.99, 95% CI = 0.986-0.995) was associated with a lower risk of mortality.

### Subanalyses of All Patients Undergoing Biopsy, Including Both Glomerular and Nonglomerular Diseases

#### Baseline characteristics

The most frequent broad diagnostic category was proliferative glomerulonephritis (n = 883, 41.9%), with nonproliferative glomerulonephritis being second most common (n = 562, 26.7%). This was true across all time periods in the study. Patients with a diagnosis of hereditary kidney disease tended to be younger, whereas patients with a deposition disease tended to be older. Patients with diabetic nephropathy, vascular disease, and tubulointerstitial disease had the lowest median estimated glomerular filtration rate (eGFR) at the time of biopsy, whereas patients with deposition diseases had the highest median urine ACR. Patients with diabetic nephropathy had the highest number of comorbidities, whereas patients with hereditary diseases had the least. The baseline characteristics of the patients are presented in Supplementary Table 6.

#### Kidney failure and mortality

Of the broad categories, the diabetic nephropathy group presented with the highest rate of kidney failure, with 74% progressing to kidney failure over a median of 4.5 years of follow-up. In contrast, of those with a diagnostic biopsy, the nonproliferative glomerulonephritis group had the lowest risk of kidney failure, with 29.9% progression to kidney failure over a median of 5.8 years of follow-up. Deposition diseases were associated with the highest risk of all-cause mortality, with 58.5% of patients dying over a median of 2.8 years of follow-up.

## Discussion

We describe the creation of a comprehensive clinicopathologic registry of biopsy-proven kidney diseases, with an emphasis on glomerular diseases. While the concept of a kidney biopsy registry is not new, with many such registries described in the literature,^13,15,26-28^ we adopted a novel method for data extraction using a natural language processing algorithm, demonstrating the feasibility of the creation of such a registry without an exhaustive chart review of potentially thousands of patients. Although natural language processing software has been used for data extraction from pathology reports in oncology,^29-31^ to our knowledge this is the first application of such software for data extraction from kidney biopsy pathology reports. Our methods could serve as a model to other centers looking to create a similar registry with high-quality data, without an in-depth manual review of potentially thousands of biopsy reports.

We describe the data linkage of pathological data from all biopsies completed in Manitoba over a 20-year period with a robust set of clinical and administrative databases that contain complete information regarding medication prescriptions, laboratory data, hospitalizations and other healthcare utilization as well as vital statistics.^32^ Such broad, yet high-quality data capture is a relatively unique feature of our registry, and only described in a handful of registries internationally.^26^ The nature of the clinical data linkage, combined with the method of data extraction from pathology reports, contributes to a highly sustainable registry requiring minimal long-term maintenance to ensure data quality. This is important, as lack of sustainability was quoted as the most common concern amongst GN registry operators in an international questionnaire.^26^

We noted several important observations in the epidemiology of glomerular disease in Manitoba in the last 2 decades. First, we observed an almost 3-fold increase in biopsy incidence over the course of the study period. This is likely multifactorial and reflects improved access to biopsy, and possibly a change in physician practice. This is not necessarily surprising, given the advancements in the understanding of the benefits of biopsy in both risk stratification and treatment selection in GN^33-36^; such an increase has been described previously.^23^ In addition, since 2002, all renal biopsy processing and reporting in Manitoba has been centralized in a core renal pathology service in one department, supported by a recognized specialist renal pathologist. This subspecialty model of pathology reporting facilitates communication between the reporting pathologist and clinicians, provides for the necessary detailed clinicopathologic correlation, and greatly improves the quality of renal biopsy reporting. This improved renal pathology service likely also contributed to the significant increase in biopsy incidence since 2002. However, our current biopsy rate appears largely similar to other regions,^37^ suggesting the current physician practice pattern is not significantly different from other centers and therefore not a likely source of bias in our registry. We also observed a striking risk of kidney failure and mortality associated with biopsy-proven infection-related glomerulonephritis, which is out of keeping with the published literature.^38,39^ This may reflect a selected population, as those with IRGN and a benign course may not be biopsied and simply managed expectantly. Nonetheless, given that our current biopsy rate is similar to other locations, this bias would likely apply to all cohorts described in the literature, and therefore further research in our cohort is required.

Compared with the published literature,^40,41^ we observed a very high kidney failure risk for patients with LN. The reasons for this are not immediately clear, but the relatively low eGFR and higher age at diagnosis raise the possibility that patients are being biopsied later in their disease course, especially considering patients with LN are typically younger than patients with lupus without LN and develop LN soon after diagnosis.^41,42^ This is further supported by the fact that most patients with lupus who have clinically apparent kidney involvement are not biopsied in our center.^43^ In patients diagnosed with IgA nephropathy, we observed a similarly striking kidney failure risk of 45% over a median 5.7 years of follow-up compared with 27.7% described in the Toronto cohort^44^ and 12% in the VALIGA cohort^45^ over similar follow-up duration. When comparing with these cohorts, our cohort was typically older, with much lower eGFR at biopsy, suggesting patients may be identified later in their disease course. With 25% of patients having an eGFR of less than 18 mL/min at biopsy, it is possible that biopsies are occurring later due to late referral. It is also possible that many patients were not biopsied earlier in their disease course due to a lack of concrete evidence regarding the use of immunosuppressive therapy for the treatment of IgA nephropathy, and that therefore a biopsy would not necessarily change management; this will likely change with the publication of more recent trials demonstrating a benefit of corticosteroids or other immunosuppressives.^46,47^ Nonetheless, more research is required to understand the factors influencing the high rate of kidney failure in this group.

Although not an immune-mediated glomerular disease, it is important to note that in our cohort, a biopsy diagnosis of diabetic nephropathy was associated with a high risk of kidney failure. Although these patients presented with low eGFR and significant albuminuria, it is also important to consider the selection bias associated with biopsy-proven diabetic nephropathy, as this typically suggests a population behaving in a manner out of keeping with typically progressing diabetic kidney disease, and thus may be at higher risk of kidney failure than those who are not biopsied. Nonetheless, it highlights an urgent need for earlier diagnosis and intervention, especially with the advent of new, efficacious therapies for preventing chronic kidney disease progression in diabetes.^48,49^ Programs such as Kidney Check, which aims to screen, triage, and treat populations at high risk of kidney disease in Canada,^50^ deserve ongoing support and funding to improve kidney failure and mortality risk due to diabetic nephropathy.

Our data are hypothesis-generating with respect to quality indicators, namely, prescription of angiotensin-converting enzyme (ACE) inhibitors and angiotensin II receptor blockers (ARBs) as well as statins. Although it is possible that many patients were not prescribed these medications due to low eGFR or side effects, such as hyperkalemia in the case of ACE inhibitors and ARBs, or myopathy in the case of statins, this finding may serve as a target for future quality improvement initiatives in our institution.

Our registry has several strengths. First, we describe a novel method of data extraction accompanied by robust data quality checks, which could serve as a model for other institutions interested in creating a glomerular diseases or kidney biopsy registry. This method contributes to the sustainability of the registry going forward and allows cases to be added as they are diagnosed. Our registry contains robust pathological data along with comprehensive clinical and administrative data, facilitating future research in patients with GN. We also have a long period of follow-up, allowing for identification of trends in disease incidence but also outcomes such as mortality and kidney failure.

Our registry also has limitations. Our study is single-center, with a relatively small number of biopsies per year as compared with other published registries. However, we captured all biopsied patients in a region with a diverse population, approximately 18% of which is Indigenous,^51^ making this a unique cohort that has yet to be described in the GN literature. In addition, kidney biopsy registries such as ours are subject to a selection bias, which affects not only disease incidences but also interpretation of outcome data, as such patients were biopsied while others were not. However, it would seem logical that patients who are biopsied have the most clinically significant disease, consistent with standard international practice.^52^ Furthermore, although our data capture is robust, data that are now indispensable in risk stratification for treatment of certain diseases, such as Oxford scores in IgA nephropathy, or phospholipase A2 receptor (PLA2R) antibody status in membranous nephropathy, are not available for many patients owing to their time of implementation in clinical care. Fortunately, these data are captured in the most recent patients and will be collected in all future patients enrolled in the registry.

## Conclusion

The Manitoba Glomerular Diseases Registry is a clinicopathologic registry created using novel data extraction techniques and capturing the spectrum of biopsy-diagnosed kidney diseases in Manitoba with a focus on glomerular disease. This registry will facilitate future research into epidemiological trends, clinical outcomes, and utilization of health services for patients with GN, including exploring the relationship among socioeconomic status, rurality, and the development of certain glomerular diseases, and will serve as a model for creation of similar registries in other institutions and health systems. Future endeavors also include creating a prospective database with biospecimens that can be linked with biopsy reports and administrative data for further understanding of disease pathways in this population.

## Supplemental Material

sj-docx-1-cjk-10.1177_20543581231178963 – Supplemental material for The Development of a Comprehensive Clinicopathologic Registry for Glomerular Diseases Using Natural Language Processing Click here for additional data file. Supplemental material, sj-docx-1-cjk-10.1177_20543581231178963 for The Development of a Comprehensive Clinicopathologic Registry for Glomerular Diseases Using Natural Language Processing by Bryce Barr, Oksana Harasemiw, Ian W Gibson, Olivier Tremblay-Savard and Navdeep Tangri in Canadian Journal of Kidney Health and Disease
