# Supplementary material for: The Development of a Comprehensive Clinicopathologic Registry for Glomerular Diseases Using Natural Language Processing
Source: Can J Kidney Health Dis. 2023 Jun 16;10:20543581231178963. doi: 10.1177/20543581231178963 (PMC10278432; doi:10.1177/20543581231178963)

**Supplementary Tables**

| **Data** | **Type** | **Possible values / Usual range of values** |
| --- | --- | --- |
| Personal Health Identification Number | numeric | 9-digit number |
| Last name | text | 1 or 2 names |
| First name | text | 1 or 2 names |
| Date of birth | numeric | DD/MM/YYYY |
| Age | numeric | Up to 3-digit number |
| Date biopsy collected / resultreceived | numeric | DD/MM/YYYY |
| Diagnosis | text | variable |
| Cores | numeric | 1-5 |
| Total glomeruli | numeric | 0-50 |
| Sclerosed glomeruli | numeric | 0-50 |
| Glomeruli with segmental sclerosis | numeric | 0-50 |
| Glomeruli with fibrinoid necrosis | numeric | 0-50 |
| Glomeruli with cellular crescents | numeric | 0-50 |
| Glomeruli with fibrocellular crescents | numeric | 0-50 |
| Glomeruli with fibrous crescents | numeric | 0-50 |
| IFTA (interstitial fibrosis and tubular atrophy) | numeric or text | 0%-100% or [mild, patchy, moderate, diffuse, significant] |
| Intimal fibrosis | text | no, yes, mild, moderate, severe |
| Vasculitis | binary | no, yes |
| Immunofluorescence | binary | no, yes |
| IgG | text | trace, 1+. 2+. 3+, +/- |
| IgA | text | trace, 1+. 2+. 3+, +/- |
| IgM | text | trace, 1+. 2+. 3+, +/- |
| AHIg | text | trace, 1+. 2+. 3+, +/- |
| C3 | text | trace, 1+. 2+. 3+, +/- |
| C1q | text | trace, 1+. 2+. 3+, +/- |
| Fibrinogen | text | trace, 1+. 2+. 3+, +/- |
| Kappa | text | trace, 1+. 2+. 3+, +/- |
| Lambda | text | trace, 1+. 2+. 3+, +/- |
| Foot Process Effacement (FPE) | text | no, yes, mild, moderate, focal, extensive, diffuse, widespread, severe |
| Subepithelial deposits | binary | no, yes |
| Subendothelial deposits | binary | no, yes |
| Intramembranous deposits | binary | no, yes |
| Mesangial deposits | binary | no, yes |
| PLA2R | binary | negative, positive |
| Oxford M/E/S/T/C | numeric | 0, 1, 2 |
| Lupus class | roman num. | I, II, III, IV, V |
| Lupus AI (activity index) | numeric | 0-24 |
| Lupus CI (chronicity index) | numeric | 0-12 |

**Table S1: Extracted pathologic data points from the biopsy reports**

**Table S2. Descriptions of MCHP databases used**

| **Database** | **Years Used** | **Description** |
| --- | --- | --- |
| Shared Health Diagnostic Services (formerly Diagnostic Services of Manitoba (DSM)) | 2002 to 2019 | Laboratory tests and results |
| Hospital Discharge Abstracts (CIHI-DAD) | 2002/2003 to 2020/2021 | Hospital admissions data |
| Manitoba Health Insurance Registry | 2002/2003 to 2020/2021 | Patient registry and provincial health insurance coverage dates |
| Medical Health Services Claims (MHSC) | January 2020 to 2020/2021 | Physician claims data |
| Drug Program Information Network (DPIN) | 2002/2003 to 2020/2021 | Drug prescriptions |
| Manitoba GN Registry | 2002 to 2019 | Information on all incident GN biopsies performed in the provinces |

**Table S3. Definitions of Dialysis and Kidney Transplantation**

| **Variable** | **Medical Health Services Claims (MHSC)** | **Hospital Discharge Abstracts (CIHI-DAD)** |
| --- | --- | --- |
| Dialysis | 2 or more codes from the following list:  9798, 9799, 9801, 9802, 9805, 9806, 9807, 9814, 9819, 9820, 9821, 3792, 3790, 3793, 3794, 3800, 3801, 3803, 3804, 3804, 9610 | -- |
| Transplantation | 1 code from either the MHSC or CIHI-DAD | |
|  | 5883 | ICD-9-CM: 556  ICD-10-CA: 1PC85, 1OK85, 556 |

**Table S4. Anatomical therapeutic chemical (ATC) codes used to classify medications**

| **Medication Type** | **ATC Codes** |
| --- | --- |
| Antihypertensives (antiadrenergic agents, diuretics, beta blocking agents, and calcium channel blockers) | C02, C03, C07, C08 |
| Angiotensin-Converting Enzyme Inhibitor / Angiotensin Receptor Blocker (ACEi / ARB) | C09 |
| Statins | C10AA, C10B |

**Table S5. Specific glomerular diseases by subcategory**

| **Proliferative glomerulonephritis** | **Non-proliferative glomerulonephritis** |
| --- | --- |
| Anti-GBM disease | C1q nephropathy |
| C3 glomerulonephritis | Chronic sclerosing glomerulopathy* |
| Cryoglobulinemic glomerulonephritis | FSGS (all subtypes with diffuse foot process effacement) |
| Dense deposit disease | Diffuse global glomerulosclerosis* |
| Fibrillary glomerulonephritis | Glomerulomegaly/obesity related glomerulopathy |
| Focal proliferative glomerulonephritis* | Idiopathic nodular glomerulosclerosis |
| IgA nephropathy | Membranous nephropathy |
| Immune-complex glomerulonephritis* | Minimal change disease |
| Infection-related glomerulonephritis | Secondary FSGS (with patchy foot process effacement) |
| Lupus nephritis |  |
| Membranoproliferative glomerulonephritis |  |
| Mesangial proliferative glomerulonephritis* |  |
| Pauci-immune glomerulonephritis |  |
| PGNMID |  |

GBM = glomerular basement membrane; PGNMID = proliferative glomerulonephritis with monoclonal immunoglobulin deposition; FSGS = focal segmental glomerulosclerosis

*Where possible, an effort was made to identify a specific diagnosis. If not possible to classify, the pathologic description (i.e focal proliferative glomerulonephritis) was used.

**Table S6. Demographic and clinical characteristics at time of biopsy for individuals who underwent a kidney biopsy in Manitoba between January 1^st^, 2002 – December 31^st^, 2019, and who had administrative health data available (N= 2103 individuals)**

|  | **Primary Diagnostic Category^1^** | | | | | | | |
| --- | --- | --- | --- | --- | --- | --- | --- | --- |
|  | Diabetic nephropathy | Proliferative glomerulonephritis | Non-proliferative glomerulonephritis | Deposition disease | Tubulointerstitial disease | Vascular Disease | Hereditary Disease | Normal/  Non-diagnostic |
| N (%) | 315 (14.9) | 883 (41.9) | 562 (26.7) | 53 (2.5) | 147 (7.0) | 68 (3.2) | 24 (1.1) | 57 (2.7) |
| Year of Diagnosis  2002-2006  2007-2012  2013-2019 | 40 (12.7)  96 (30.5)  179 (56.8) | 157 (17.8)  263 (29.8)  463 (52.4) | 102 (18.2)  181 (32.2)  279 (49.6) | 10 (18.9)  12 (22.6)  31 (58.5) | 31 (21.1)  51 (34.7)  65 (44.2) | 11 (16.2)  14 (20.6)  43 (63.2) | 10 (41.7)  *suppressed*  *suppressed* | 10 (17.5)  12 (21.1)  35 (61.4) |
| Age, years | 53.5 ± 12.7 | 48.4 ± 17.6 | 53.2 ± 16.3 | 67.7 ± 10.4 | 59.2 ± 15.6 | 57.5 ± 17.8 | 41.8 ± 11.1 | 51.4 ± 17.7 |
| Female sex | 141 (44.8) | 437 (49.5) | 209 (37.2) | 22 (41.5) | 58 (39.5) | 33 (48.5) | 14 (58.3) | 22 (38.6) |
| eGFR, mL/min/1.73 m^2^ | 20.0 (12.0, 24.0) | 31.5 (14.5, 67.0) | 48.5 (25.0, 87.0) | 43.0 (21.5, 71.5) | 20.0 (9.0, 43.5) | 20.0 (8.0, 33.0) | 98.5 (81.5, 108.0) | 61.5 (33.0, 99.0) |
| Urine Albumin-to-Creatinine Ratio, mg/mmol | 407.1 (233.9, 677.3) | 168 (85.7, 366.1) | 294.8 (138.0, 539.6) | 438.1 (234.2, 951.2) | 38.0 (8.4, 123.8) | 38.6 (7.9, 167.9) | 26.0 (3.2, 100.2) | 25.8 (2.7, 136.3) |
| Dwelling location  Rural  Urban | 138 (44.1)  175 (55.9) | 388 (44.0)  493 (56.0) | 199 (35.5)  361 (64.5) | 13 (24.5)  40 (75.5) | 58 (39.5)  89 (60.5) | 20 (29.4)  48 (70.6) | 8 (33.3)  16 (66.7) | 14 (24.6)  43 (75.4) |
| Socioeconomic Status  1 (Lowest)  2  3  4  5 (Highest) | 134 (42.8)  73 (23.3)  37 (11.8)  41 (13.1)  28 (9.0) | 283 (32.1)  178 (20.2)  139 (15.8)  174 (19.8)  107 (12.2) | 138 (24.6)  113 (20.2)  94 (16.8)  112 (20.0)  103 (18.4) | 12 (22.6)  12 (22.6)  7 (13.2)  10 (18.9)  12 (22.6) | 33 (22.5)  20 (13.6)  28 (19.1)  41 (27.9)  25 (17.0) | 18 (26.5)  13 (19.1)  17 (25.0)  9 (13.2)  11 (16.2) | *suppressed* | 9 (15.8)  12 (21.1)  18 (31.6)  8 (14.0)  10 (17.5) |
| Medications |  |  |  |  |  |  |  |  |
| Anti-Blood Pressure Medications *(not including ACEi/ARBs)* | 295 (93.7) | 572 (64.8) | 431 (76.7) | 40 (75.5) | 82 (55.8) | 56 (82.4) | *suppressed* | 22 (38.6) |
| ACEi/ARBs | 197 (62.5) | 438 (49.6) | 402 (71.5) | 21 (39.6) | 26 (17.7) | 33 (48.5) | 13 (54.2) | 25 (43.9) |
| Statins | 190 (60.3) | 195 (22.1) | 257 (45.7) | 16 (30.2) | 35 (23.8) | 27 (39.7) | *suppressed* | 16 (28.1) |
| Comorbidities* | 4.0 (2.0, 5.0) | 1.0 (0, 3.0) | 1.0 (0, 2.0) | 2.0 (1.0, 3.0) | 2.0 (1.0, 4.0) | 1.0 (0, 3.0) | 0.5 (0, 1.0) | 1.0 (0, 3.0) |
| Follow-up time (years)** | 4.5 (2.3, 7.3) | 5.4 (2.8, 9.9) | 5.8 (3.1, 10.5) | 2.8 (1.2, 6.3) | 4.3 (1.7, 8.3) | 3.9 (1.9, 7.0) | 6.6 (4.4, 14.5) | 4.6 (2.4, 9.5) |
| All-cause mortality | 162 (51.4) | 248 (28.1) | 122 (21.7) | 31 (58.5) | 70 (47.6) | 22 (32.4) | 0 | 13 (22.8) |
| Kidney Failure*** | 233 (74.0) | 423 (47.9) | 168 (29.9) | 22 (41.5) | 78 (53.1) | 40 (58.8) | *suppressed* | 12 (21.1) |

Notes. ^1^Indivdiuals may be included in more than one category if they had multiple biopsies where the subsequent biopsy revealed a different primary diagnosis.
Data presented as N (%) for categorical variables, mean ± standard deviation for normally distributed continuous variables, and median (interquartile range) for non-normally distributed continuous variables, as appropriate. eGFR = estimated Glomerular Filtration Rate; ACEi = Angiotensin-Converting Enzyme Inhibitor; ARB = Angiotensin Receptor Blocker.
Labs values were assessed ± 3 months of biopsy, medications were assessed within 6 months post-biopsy
*Comorbid conditions were based on the weighted Charlson Comorbidity Index and were drawn from data collected through medical claims and hospital admission data during the 3 years prior to the date of biopsy.
**Individuals were followed until they either: reached the end of the study period (March 31^st^, 2021), lost provincial health coverage, migrated from the province, died, or had a second follow-up biopsy that resulted in a different primary diagnosis.
***Kidney Failure defined as requiring dialysis or kidney transplantation.
Cell sizes <6 are suppressed to protect individual anonymity. Urine ACR values includes urine protein-to-creatinine ratio tests converted to urine ACR using the equation developed by Weaver et al. 2020.

**Table S7. Hazard ratios and 95% confidence intervals for the association between primary diagnosis and the outcomes of mortality and kidney failure**

|  | **Mortality ^a^** | **Mortality ^b^** | **Kidney Failure ^c^** | **Kidney Failure ^d^** |
| --- | --- | --- | --- | --- |
| Diabetic Nephropathy | **2.93 (2.18, 3.94)** | **2.45 (1.79, 3.36)** | **4.55 (3.52, 5.87)** | **2.09 (1.61, 2.71)** |
| Proliferative Glomerulonephritis | 1.28 (0.97, 1.70) | **1.65 (1.23, 2.22)** | **1.79 (1.41, 2.27)** | 1.27 (0.99, 1.63) |
| Non-proliferative Glomerulonephritis | Reference Category | Reference Category | Reference Category | Reference Category |
| Deposition Disease | **4.04 (2.44, 6.69)** | **2.45 (1.48, 4.07)** | **2.23 (1.27, 3.91)** | **2.12 (1.20, 3.73)** |
| Tubulointerstitial Disease | **2.52 (1.70, 3.78)** | **2.98 (1.89, 4.68)** | **1.68 (1.06, 2.64)** | 1.39 (0.86, 2.24) |
| Vascular Disease | **2.57 (1.53, 4.30)** | **2.57 (1.48, 4.45)** | **2.50 (1.43, 4.38)** | **2.64 (1.48, 4.70)** |
| Hereditary Disease | --- | --- | 0.48 (0.12, 1.95) | 1.76 (0.43, 7.17) |
| Normal/Non-Diagnostic Biopsy Findings | 1.49 (0.72, 3.10) | 2.11 (1.01, 4.41) | 0.81 (0.38, 1.75) | 1.40 (0.65, 3.05) |
| Age at biopsy |  | **1.05 (1.04, 1.06)** |  | **0.95 (0.95, 0.96)** |
| Sex ^1^ |  | **1.24 (1.01, 1.51)** |  | **1.42 (1.30, 1.54)** |
| eGFR |  | **0.99 (0.98, 0.99)** |  | **0.98 (0.97, 0.98)** |
| Log Urine Albumin-to-Creatinine Ratio (ACR) |  | **1.36 (1.25, 1.49)** |  | **1.42 (1.19, 1.69)** |
| **AIC** | 5321.15 | 5037.19 | 6991.22 | 6369.26 |
| **AUC** | 0.60 | 0.76 | 0.64 | 0.83 |

*: Reference category is Non-proliferative Glomerulonephritis

A: Model includes Primary Diagnosis Category (excluding Hereditary Disease category)

B: Model includes Primary Diagnosis Category (excluding Hereditary Disease category), age, sex, eGFR, urine ACR

C: Model includes all Primary Diagnosis Categories
D: Model includes all Primary Diagnosis Categories, age, sex, eGFR, urine ACR

1: Reference category is Females

eGFR = estimated Glomerular Filtration Rate

AIC= Akaike information criterion; AUC = Area Under the Curve

N= 1456 for mortality outcome; N= 1337 for kidney failure outcome
Excluding those with >1 primary diagnosis

Supplemental Figure 1: Kaplan-Meier analysis of kidney failure among individuals with biopsy-proven GN in Manitoba


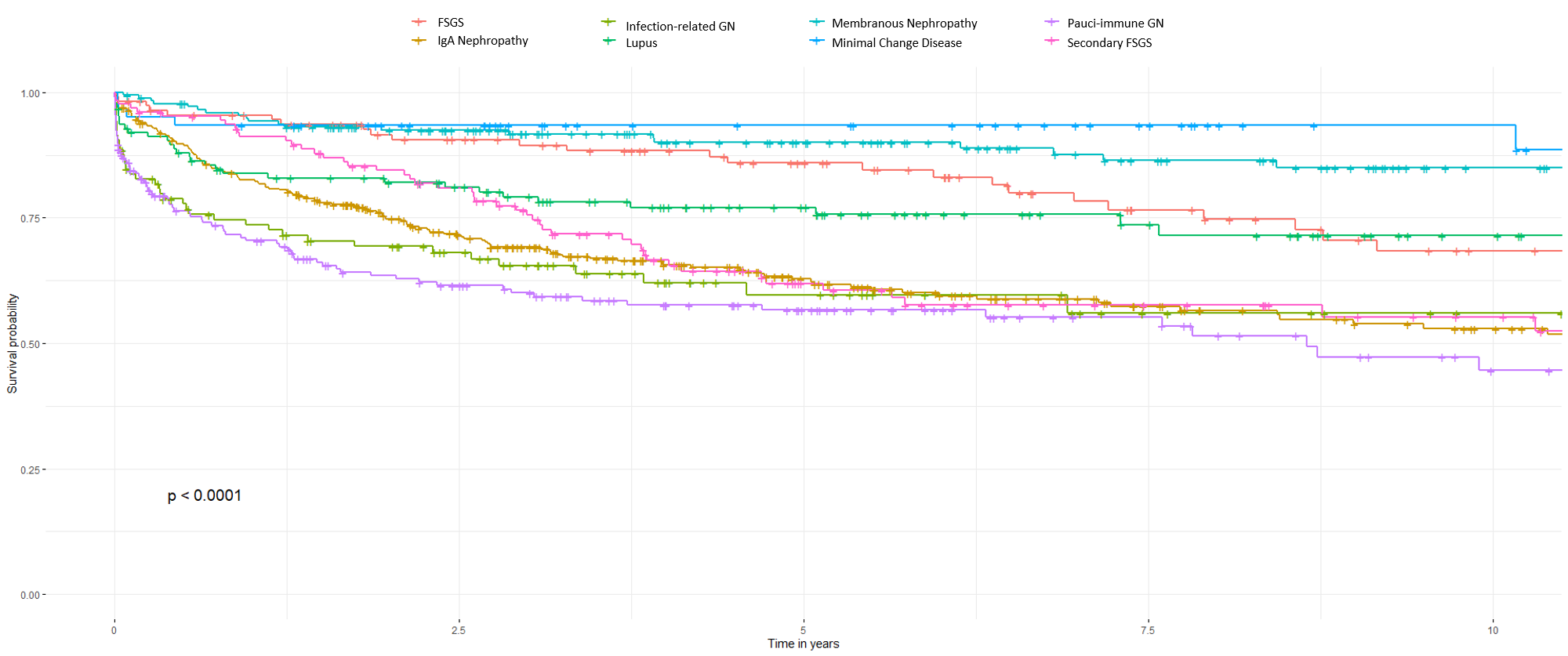


Supplemental Figure 2: Kaplan-Meier analysis of mortality among individuals with biopsy-proven GN in Manitoba


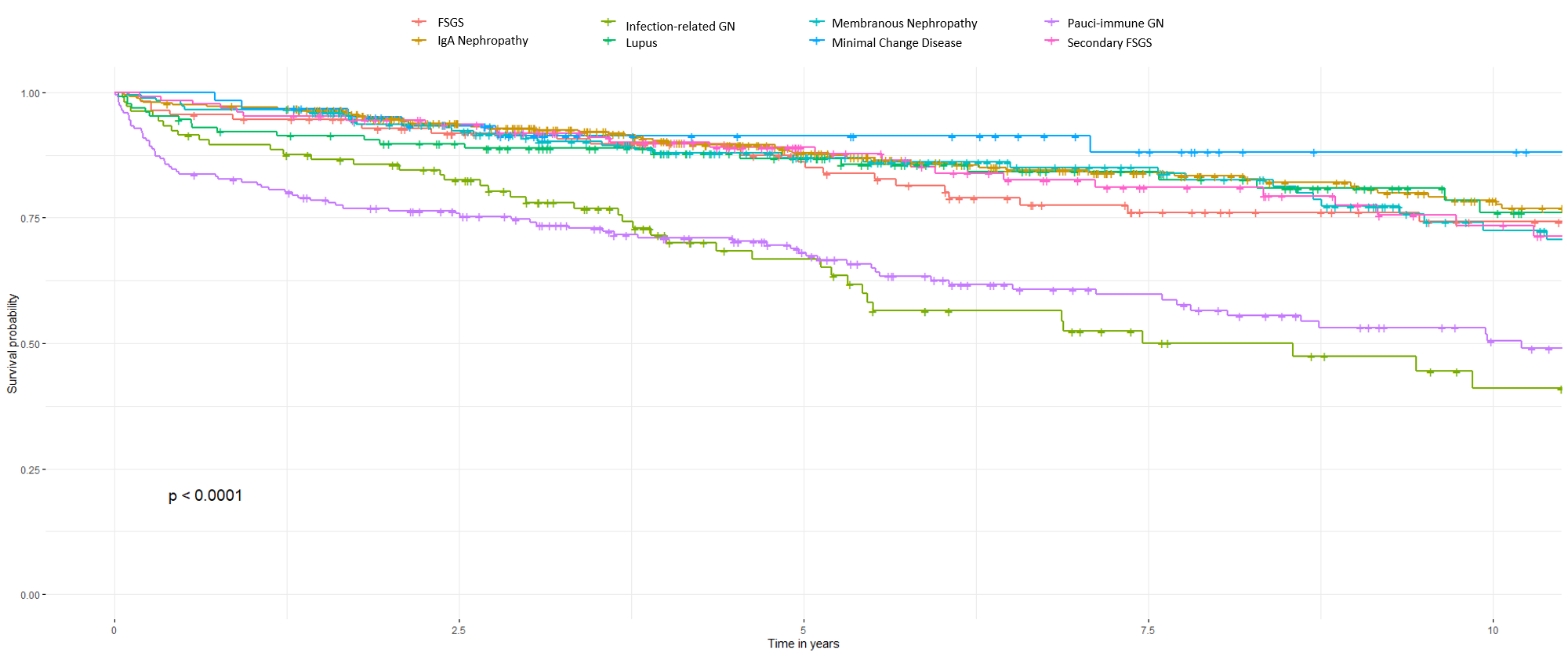

Supplement: sj-docx-1-cjk-10.1177_20543581231178963 – Supplemental material for The Development of a Comprehensive Clinicopathologic Registry for Glomerular Diseases Using Natural Language Processing [file sj-docx-1-cjk-10.1177_20543581231178963.docx]
